# Tumor-Derived Interleukin-1 Promotes Lymphangiogenesis and Lymph Node Metastasis through M2-Type Macrophages

**DOI:** 10.1371/journal.pone.0099568

**Published:** 2014-06-12

**Authors:** Kosuke Watari, Tomohiro Shibata, Akihiko Kawahara, Ken-ichi Sata, Hiroshi Nabeshima, Ai Shinoda, Hideyuki Abe, Koichi Azuma, Yuichi Murakami, Hiroto Izumi, Takashi Takahashi, Masayoshi Kage, Michihiko Kuwano, Mayumi Ono

**Affiliations:** 1 Department of Pharmaceutical Oncology, Graduate School of Pharmaceutical Sciences, Kyushu University, Fukuoka, Japan; 2 Department of Diagnostic Pathology, Kurume University Hospital, Kurume, Japan; 3 Department of Internal Medicine, Division of Respirology, Neurology, and Rheumatology, Kurume University School of Medicine, Kurume, Japan; 4 St. Mary's Hospital, Kurume, Japan; 5 Department of Occupational Pneumology, Institute of Industrial Ecological Sciences, University of Occupational and Environmental Health, Kitakyushu, Japan; 6 Division of Molecular Carcinogenesis, Center for Neurological Diseases and Cancer, Nagoya University Graduate School of Medicine, Nagoya, Japan; 7 Laboratory of Molecular Cancer Biology, Graduate School of Pharmaceutical Sciences, Kyushu University, Fukuoka, Japan; Istituto Superiore di Sanità, Italy

## Abstract

Tumors formed by a highly metastatic human lung cancer cell line are characterized by activated signaling via vascular endothelial growth factor (VEGF)-C through its receptor (VEGFR-3) and aggressive lymph node metastasis. In this study, we examined how these highly metastatic cancers acquired aggressive lymph node metastasis. Compared with their lower metastatic counterparts, the highly metastatic tumors formed by this cell line expressed higher amounts of interleukin (IL)-1α, with similarly augmented expression of IL-1α and IL-1β by tumor stromal cells and of VEGF-A and VEGF-C by tumor-associated macrophages. These tumor-associated macrophages were mainly of the M2 type. Administration of a macrophage-targeting drug suppressed the production of these potent angiogenic and lymphangiogenic factors, resulting in decreased tumor growth, angiogenesis, lymphangiogenesis, and lymph node metastasis. In Matrigel plug assays, the highly metastatic cells formed tumors that were extensively infiltrated by M2-type macrophages and exhibited enhanced angiogenesis and lymphangiogenesis. All of these responses were suppressed by the IL-1 receptor (IL-1R) antagonist anakinra. Thus, the IL-1α-driven inflammatory activation of angiogenesis and lymphangiogenesis seems to provide a highly metastatic tumor microenvironment favorable for lymph node metastasis through cross-talk with macrophages. Accordingly, the IL-1R/M2-type macrophage axis may be a good therapeutic target for patients with this form of lung cancer.

## Introduction

The treatment of cancer patients is often complicated by tumor angiogenesis and lymphangiogenesis, which are closely associated with tumor metastasis and growth [1]–[3]. Identifying the molecules involved in these processes could help to advance therapeutic strategies for cancer patients. Both angiogenesis and lymphangiogenesis are exacerbated in tumors by the up-regulation of chemokines, growth factors, proteolytic enzymes, and prostaglandins in response to inflammatory stimuli [4]–[6]. In fact, human malignancies are often initiated and promoted by inflammation, in close association with angiogenesis [4]–[6] and lymphangiogenesis [2], [7], while the recruitment of macrophages and neutrophils to the tumor microenvironment activates cells that support cancer progression [6], [8]–[11]. In the cornea, inflammatory cytokines such as interleukin (IL)-1α and IL-1β induce angiogenesis and lymphangiogenesis by enhancing the expression of angiogenic and lymphangiogenic factors in a sequence of events that can be blocked by macrophage depletion [12]–[15]. Clinical studies have also demonstrated a close association between infiltration of tumor-associated macrophages (TAMs) and poor prognosis in patients with various human malignancies [16], [17], suggesting that elevated inflammatory responses in the tumor microenvironment are important for malignant progression. It has been proposed that TAMs are composed of functionally different populations of angiogenesis-, metastasis-, and inflammation-supporting macrophages, thereby allowing these cells to influence tumor development [11], [18].

The human lung cancer cell line NCI-H460-LNM35 (LNM35) is highly metastatic compared with its lower metastatic counterpart N15, and the cells have a propensity to cause lymph node metastases following subcutaneous or orthotopic injection in mice [19]. Previous studies examining the mechanism(s) underlying the lung and lymph node metastasis of these cells noted the following findings. First, cyclooxygenase 2 (COX2) expression and invasion/motility *in vitro* were higher in LNM35 than in N15 cells. In a xenograft model *in vivo*, lung cancer metastasis was blocked by COX2 inhibitors [20], indicating a role for prostaglandin pathways, as well as inflammatory activation, in metastasis. Second, LNM35 cells expressed slightly higher amounts of the potent lymphangiogenic factor vascular endothelial growth factor (VEGF)-C, together with activation of its cognate receptor (VEGFR-3) [21], [22]. In highly metastatic tumors treated with soluble VEGFR-3 and a receptor tyrosine kinase inhibitor, lymphangiogenesis, lymph node metastasis, and tumor growth were blocked [21]–[23]. Third, the angiopoietin/Tie2 pathway was activated in highly metastatic cells, and an angiopoietin 2-blocking antibody suppressed lung and lymph node metastasis [24]. Taken together, these findings suggest that the induction of lung and lymph node metastasis, lymphangiogenesis, and tumor growth by highly metastatic lung cancer cells is mediated by enhanced expression of COX2, VEGFR-3-driven signaling, and angiopoietin.

The hypothesis underlying the present study was that increased inflammatory responses mediated by IL-1 and other stimuli are crucial for the highly lymphangiogenic and angiogenic potential of lung cancer cells. In recent work, we showed that IL-1-driven inflammatory signaling promotes tumor angiogenesis and infiltration of TAMs by gastric cancer cells [25]. Here, we investigated whether inflammatory stimuli play a role in the mechanism by which lung cancer cells acquire a high metastatic potential. Our results show that IL-1-driven inflammatory signaling leads to recruitment of M2-type macrophages through induction of CXC chemokines by cancer cells, thereby promoting tumor growth and lymph node metastasis via tumor lymphangiogenesis and angiogenesis. Accordingly, we discuss whether targeting of IL-1/IL-1 receptor (IL-1R) and/or tumor-supporting M2-type macrophages will offer useful therapeutic strategies for inhibition of tumor growth and lymph node metastasis, through suppression of lymphangiogenesis and angiogenesis by lung cancer cells.

## Materials and Methods

### Ethics statement

All animal experiments were approved by the Ethics of Animal Experiments Committee at Kyushu University Graduate School of Medical Sciences. Male KSN nude mice were purchased from Japan SLC, Inc. (Shizuoka, Japan) and Male C57BL/6 mice were purchased from CLEA (Saga, Japan), and housed in microisolator cages maintained under a 12-hr light/dark cycle. Water and food were supplied ad libitum. Animals were observed for signs of tumor growth, activity, feeding, and pain in accordance with the guidelines of the Harvard Medical Area Standing Committee on Animals.

### Cell culture and reagents

LNM35 and N15 cell lines were established as described previously [19], [20]. The cells were maintained in RPMI 1640 medium supplemented with 10% FBS and incubated in a humidified atmosphere of 5% CO_2_ at 37°C. LLC/IL-1β cell line and the control LLC/neo cell line were donated by Y. Saijo and T. Nukiwa (Tohoku University) and cultured in Dulbeccos's modified Eagles medium with 10% FBS [13], [14]. Anakinra, used in the treatment of rheumatoid arthritis, was kindly provided by Swedish Orphan Biovitrum Sverige AB. Anti-β-actin and anti-IL-1RI antibodies were purchased from Abcam (Cambridge, UK). The anti-COX2 antibody was from Takara (Shiga, Japan). Anti-IL-1α antibodies, anti-human and mouse VEGF-A, and anti-human and mouse VEGF-C were from Santa Cruz Biotechnology (Santa Cruz, CA, USA); the anti-mouse CD31 antibody was from BMA Biomedicals (Augst, Switzerland); anti-mouse F4/80 was from AbD Serotec (Kidlington, UK); anti-mouse LYVE-1 was from ReliaTech (Wolfenbüttel, Germany); CF488A donkey anti-rat IgG and CF594 donkey anti-rabbit IgG were from Biotium (Hayward, CA); Alexa-594 chicken anti-goat IgG was from Invitrogen Corporation (Carlsbad, CA); SB225002 was from Calbiochem (Darmstadt, Germany), and both the CXCR2 neutralizing antibody and the anti-mouse Gr-1 were from R&D Systems (Minneapolis, MN). The anti-GAPDH antibody used in western blot analysis was from Travigen (Gaithersburg, MD).

### Nude mouse xenograft models

Approximately 1.0×10^6^ LNM35 or N15 cells in 100 µL of serum-free RPMI 1640 medium were implanted into the subcutaneous tissue of the right abdominal wall of male KSN/slc mice (6–7 weeks old; one tumor per mouse; n = 6 mice per group). Tumor size was measured with calipers, and tumor volumes (mm^3^) were calculated as follows: volume = length×width^2^×0.5. The mice were killed after 35 days and their lungs and axillary lymph nodes, as well as the tumors, were collected. Tumor samples were stored at −80°C for protein analysis or fixed immediately in 10% paraformaldehyde overnight at 4°C and then processed for further histological analysis.

In separate experiments, approximately 1.0×10^6^ LNM35 cells were subcutaneously implanted into male KSN/slc mice (one tumor per mouse). Either anakinra (2.5 or 5 mg/mouse/day) was injected subcutaneously daily or Cl_2_MDP-LIP was injected through a tail vein via a 27-gauge needle once every 3 days. The mice were killed 35 days later; the tissues were collected and processed as described above.

### Preparation of Cl_2_MDP-LIP

Cl_2_MDP-LIP was prepared as described previously [13]. Cholesterol (11 mg) and phosphatidylcholine (75 mg) were mixed with 10 mL of 0.7 M Cl_2_MDP solution and the mixture was sonicated gently. The resulting liposomes were washed three times to eliminate free drug. Empty liposomes were prepared as a control under the same conditions, but using PBS instead of Cl_2_MDP.

### Immunohistochemical (IHC) analysis

Tissue sections were immunohistochemically stained with anti-CD31 (PECAM-1), anti-LYVE-1, anti-F4/80, and anti-Gr-1 antibodies using the peroxidase method (Histofine SABPO kit; Nichirei, Tokyo, Japan). In all tissue samples, the mean number of infiltrating macrophages and neutrophils, the microvascular density, and the lymphatic vessel density were calculated from five hotspots. All counts were performed by three independent observers. Sections of the axillary lymph nodes were stained with hematoxylin and eosin (H&E).

### Immunofluorescence

Tumors were snap frozen in OCT (Sakura Finetechnical Co. Ltd; Tokyo, Japan), and 5-µm sections were then cut, air dried, and fixed for 3 min in cold acetone. Tissues were stained overnight at 4°C with two primary antibodies, either polyclonal rabbit anti-human and mouse VEGF-A (1/200) or polyclonal goat anti-human and mouse VEGF-C (1/200), followed by rat anti-mouse F4/80 antibody. The following day, the slides were washed and then labeled with secondary antibody conjugates for 30 min at room temperature. The secondary antibodies used against VEGF-A, VEGF-C, and F4/80 were CF594 donkey anti-rabbit IgG (1/500), Alexa-594 chicken anti-goat IgG (1/250), and CF488A donkey anti-rat IgG (1/500), respectively.

### Determination of cytokines by ELISA

The concentrations of human IL-1α, IL-1β, VEGF-A, VEGF-C, MCP-1/CCL2, Groα/CXCL1, ENA-78/CXCL5, IL-8/CXCL8, and IL-6 in the homogenized supernatants of mouse xenograft tumors and in conditioned medium were measured using commercially available ELISA kits (R&D Systems). When the cells reached subconfluence, the medium was replaced with serum-free RPMI and the cells were incubated for another 24 h. The results, normalized for the number of cells, are reported as picograms (pg) of growth factor/10^5^ cells/24 h. Tumor tissue obtained from mice was homogenized in T-PER tissue protein extraction reagent containing 1 mM EDTA, 0.1 mM Na_3_VO_4_, 1 mM PMSF, 10 µg aprotinin/mL, and 10 µg leupeptin/mL, and centrifuged at 13,000 rpm for 10 min.

### qRT-PCR

Total RNA was isolated from cell cultures and tumors using Isogen (Nippon Gene Co. Ltd., Tokyo, Japan), according to the manufacturer's instructions. The RNA concentration was assessed spectrophotometrically at 260 nm. RNA was reverse-transcribed from random hexamers using AMV reverse transcriptase (Promega, Madison, WI). qRT-PCR was performed using the Real-Time PCR system 7300 (Applied Biosystems, Foster City, CA) in reaction mixtures containing cDNA, the primer pairs, the dual-labeled fluorogenic probe, and TaqMan Universal PCR Master Mix (Applied Biosystems, Santa Clara, CA). Primer pairs and probes were obtained from Applied Biosystems. The thermal-cycle conditions were 95°C for 10 min, then 95°C for 15 s, and 60°C for 1 min, alternating for 40 cycles. Relative gene expression in each sample was determined using the following formula: 2∧(-delta Ct) = 2∧[Ct(GAPDH)-Ct(target)]. Target-gene expression was normalized to glyceraldehyde 3-phosphate dehydrogenase (GAPDH) levels.

### Preparation of peritoneal macrophages

Peritoneal cells were obtained 4 days after the intraperitoneal injection of 2 mL of 3% thioglycollate medium into each mouse. The cells were suspended in RPMI 1640 medium and incubated for 120 min at 37°C in a CO_2_ incubator to allow them to adhere to the plates. The medium was then withdrawn and non-adherent cells were removed by washing the plates twice with pre-warmed PBS.

### Co-culture of mouse peritoneal macrophages with human lung cancer cells

Macrophages (2.0×10^5^) and either N15 or LNM35 cells (2.5×10^4^) were seeded in 10% FBS RPMI 1640 with or without anakinra (50 ng/mL) and incubated for 72 h. Total RNA was then extracted and processed as described above.

### Mouse peritoneal macrophage migration assay

The migration assay was performed using a multiwell chamber as the outer chamber and 8-µm pore-size polycarbonate filters (BD Biosciences, Bedford, MA) as the inner chamber. N15 or LNM35 cells were seeded in a 24-well plate (5.0×10^4^ per well), cultured for 24 h, and then transferred to serum-free medium with or without anakinra. Mouse peritoneal macrophages were suspended in serum-free medium with or without SB225002 or CXCR2 neutralizing antibody and seeded (3.0×10^5^ cells per inner chamber) on polycarbonate filters. After 24 h at 37°C, the medium was withdrawn from the inner chamber; the cells from the upper surface of the filters were removed with cotton swabs. Cells on the lower surface were fixed with methanol, stained with Giemsa, and counted at a magnification of 200× in four fields per chamber. The results are presented as the means determined in the four chambers.

### Western blot analysis

The cells were rinsed with ice-cold PBS and lysed in buffer (pH 8.0) comprising 50 mM Tris-HCl, 250 mM NaCl, 0.3% NP-40, 1 mM EDTA, 10% glycerol, 0.1 mM Na_3_VO_4_, 50 mM NaF, 1 mM phenylmethylsulfonyl fluoride (PMSF), 10 µg aprotinin/mL, and 10 µg leupeptin/mL. The cell lysates were subjected to SDS-PAGE and transferred to Immobilon membranes (Millipore, Bedford, MA, USA). Each membrane was then incubated with blocking solution followed by primary antibody. Antibody detection was carried out using an enhanced chemiluminescence system (Amersham Biosciences Corp., Piscataway, NJ, USA). The intensity of the luminescence was quantified using a charge-coupled device camera combined with an image-analysis system (LAS-4000 mini; GE Healthcare, Buckinghamshire, UK).

### Matrigel plug assay

Mice were injected subcutaneously at the abdominal midline with 0.5 mL of growth factor-reduced Matrigel matrix (BD Bioscience) supplemented with N15 cells or LNM35 cells (1×10^6^) with or without anakinra (1 µg/mL), or LLC/neo cells or LLC/IL-1β cells (5×10^4^). After 10 days, the Matrigel plugs were removed and snap frozen in OCT. The tissue blocks were used to cut 5-µm sections, which were first air dried and then fixed for 3 min in cold acetone. The sections were stained with anti-CD31, anti-LYVE-1, and anti-F4/80 antibodies.

### Macrophage from tumors and Matrigel plugs

CD11b+ cells were isolated from mouse xenograft tumors or Matrigel plugs by magnetic sorting using CD11b MicroBeads (Miltenyi Biotec GmbH, Bergisch-Gladbach, Germany). In brief, tissues minced in PBS were incubated in collagenase L (Nitta Gelatin, Osaka, Japan) and DNase I (Roche Diagnostics GmbH, Mannheim, Germany) at final concentrations of 0.5% and 20 unit/mL. The mixture was incubated for 1 h at 37°C under gentle agitation. Digestion was stopped using fetal bovine serum, after which the cell suspension was washed and then passed through a 70-µm-mesh nylon screen. The cells were incubated with CD11b MicroBeads for 15 min at 4°C and loaded onto a MIDIMACS column (Miltenyi Biotec GmbH) according to the manufacturer's instructions. Isolated CD11b+ cells from xenografts or Matrigel were used as TAMs for further experiments. For cell surface staining, single-cell suspensions were incubated with fluorescein isothiocyanate–conjugated anti-CD11b monoclonal antibody (BD Bioscience) and phycoerythrin-conjugated anti-F4/80 monoclonal antibody (eBioscience, Inc., San Diego, CA) for 15 min at 4°C. The stained cells were run on a FACSCalibur flow cytometer (BD Biosciences). The data were analyzed using the CellQuest software program (BD Biosciences).

## Results

### Highly metastatic cancer cells show enhanced macrophage and neutrophil infiltration and lymph node metastasis in vivo

**Figure 1:**
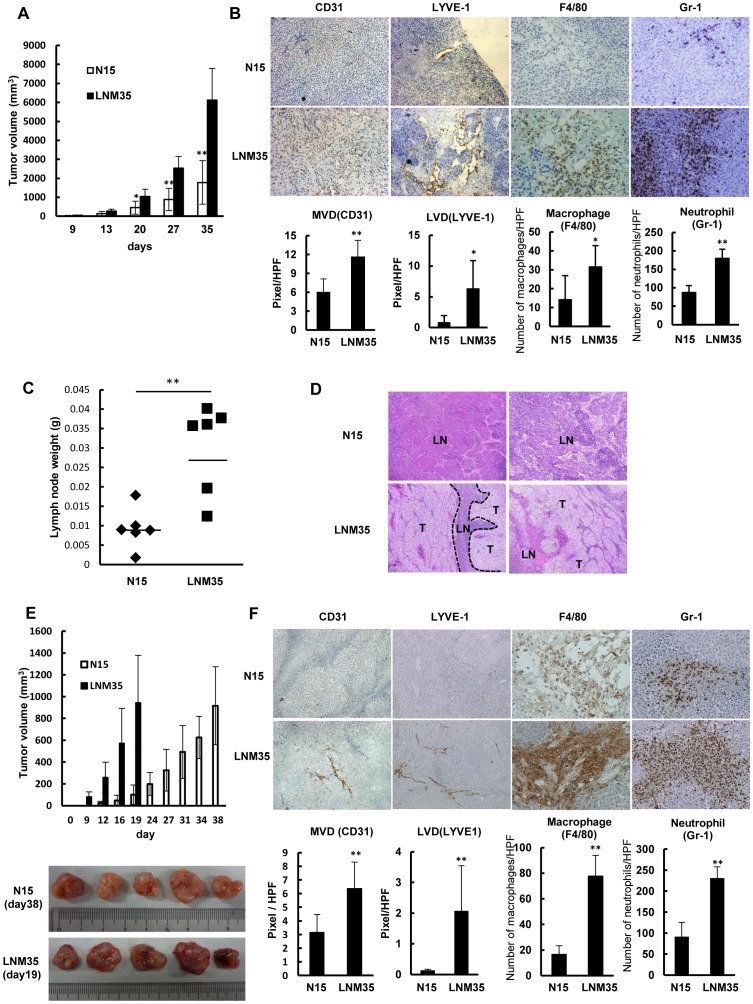
Highly metastatic LNM35 tumors are highly angiogenic and lymphangiogenic and rich in macrophages. (A) Comparison of tumor growth rates between N15 and LNM35 cells inoculated subcutaneously at day 0. Tumor growth rates were significantly different (**p<0.01, n = 6). (B) IHC analysis using antibodies against vascular endothelium (CD31) and lymphatic vessels (LYVE-1), a macrophage-specific antibody (F4/80), and a neutrophil-specific antibody (Gr-1). N15 and LNM35 tumors were quantitatively analyzed, scoring five areas in each tumor section for microvascular density (MVD), lymphatic vessel density (LVD), F4/80-positive cells, and Gr-1-positive cells; *p<0.05 and ** *p*<0.01. (C) Comparison of lymph node weights between N15 and LNM35 tumors on day 35 after subcutaneous inoculation (**p<0.01, n = 6). (D) H&E staining of lymph node samples from mice subcutaneously injected with N15 and LNM35 cells (n = 2 each); T: tumor, LN: lymph node. (E) Comparison of tumor growth rates following the subcutaneous inoculation of N15 and LNM35 cells at day 0. N15 tumors at day 38 and LNM35 tumors at day19 were of similar size and were further analyzed. (F) IHC analysis using antibodies against vascular endothelium (CD31), lymphatic vessels (LYVE-1), macrophages (F4/80), and neutrophils (Gr-1). Five N15 and LNM35 tumors were quantitatively analyzed, scoring five areas in each tumor section for MVD, LVD, F4/80-positive cells, and Gr-1-positive cells. ** *p*<0.01.

The highly metastatic cancer cell line NCI-H460-LNM35 (LNM35) and its lower metastatic counterpart NCI-H460-N15 (N15) were originally established from metastatic foci in lymph nodes of mice after subcutaneous injection of cells from the human lung cancer cell line NCI-H460 [19], [20]. Under basal growth conditions, the two cell lines have similar proliferation rates, with a doubling time of ∼16 h (data not shown). However, in an *in vivo* xenograft model, their tumor growth rates differed markedly (Figure 1A). Therefore, we used these two cell lines as a model system to compare the angiogenesis, lymphangiogenesis, macrophage and neutrophil infiltration, and lymph node weight in the respective tumors evaluated at 35 days after subcutaneous injection of these cells (Figure 1B, C). IHC analysis revealed important differences between N15 and LNM35 tumors in the development of hemangiogenic microvessels (CD31+) and lymphatic vessels (LYVE-1+), and the extent of macrophage (F4/80+) and neutrophil (Gr-1+) infiltration (Figure 1B). Quantitative analyses confirmed that the angiogenesis, lymphangiogenesis, and macrophage and neutrophil infiltration were significantly greater in the LNM35 tumors (Figure 1B). Consistent with these findings, the lymph nodes of mice bearing LNM35 tumors were more than three-fold larger and heavier than the lymph nodes of mice bearing N15 tumors and comprised human cancer cells, stromal cells, and lymphatic vessels (Figure 1C). Furthermore, the incidence of lymph node metastasis was increased in all mice with subcutaneous implantations of LNM35 cells, compared with mice implanted with N15 cells. The lymph nodes of the LNM35-implanted mice were almost completely occupied by cancer cells (Figure 1D).

As the two cell lines differed in their tumor growth rates *in vivo*, we next examined the angiogenesis, lymphangiogenesis, and macrophage infiltration in N15 and LNM35 tumors of similar sizes obtained at days 38 and 19, respectively (Figure 1E). The LNM35 tumors contained more blood and were thus redder than the N15 tumors (Figure 1E), reflecting the previously determined differences with respect to angiogenesis, lymphangiogenesis, and macrophage and neutrophil infiltration (Figure 1F). Quantitative analyses showed significant (**p<0.01) differences in the microvascular density, lymphatic vessel density, and numbers of infiltrating macrophages and neutrophils. These results suggest that LNM35 cells have a greater intrinsic potential than N15 cells for angiogenesis and lymphangiogenesis, irrespective of the differences in tumor growth.

### Expression of IL-1α, VEGF-C, and CXC chemokines is enhanced in highly metastatic cancer cells in vitro

**Figure 2:**
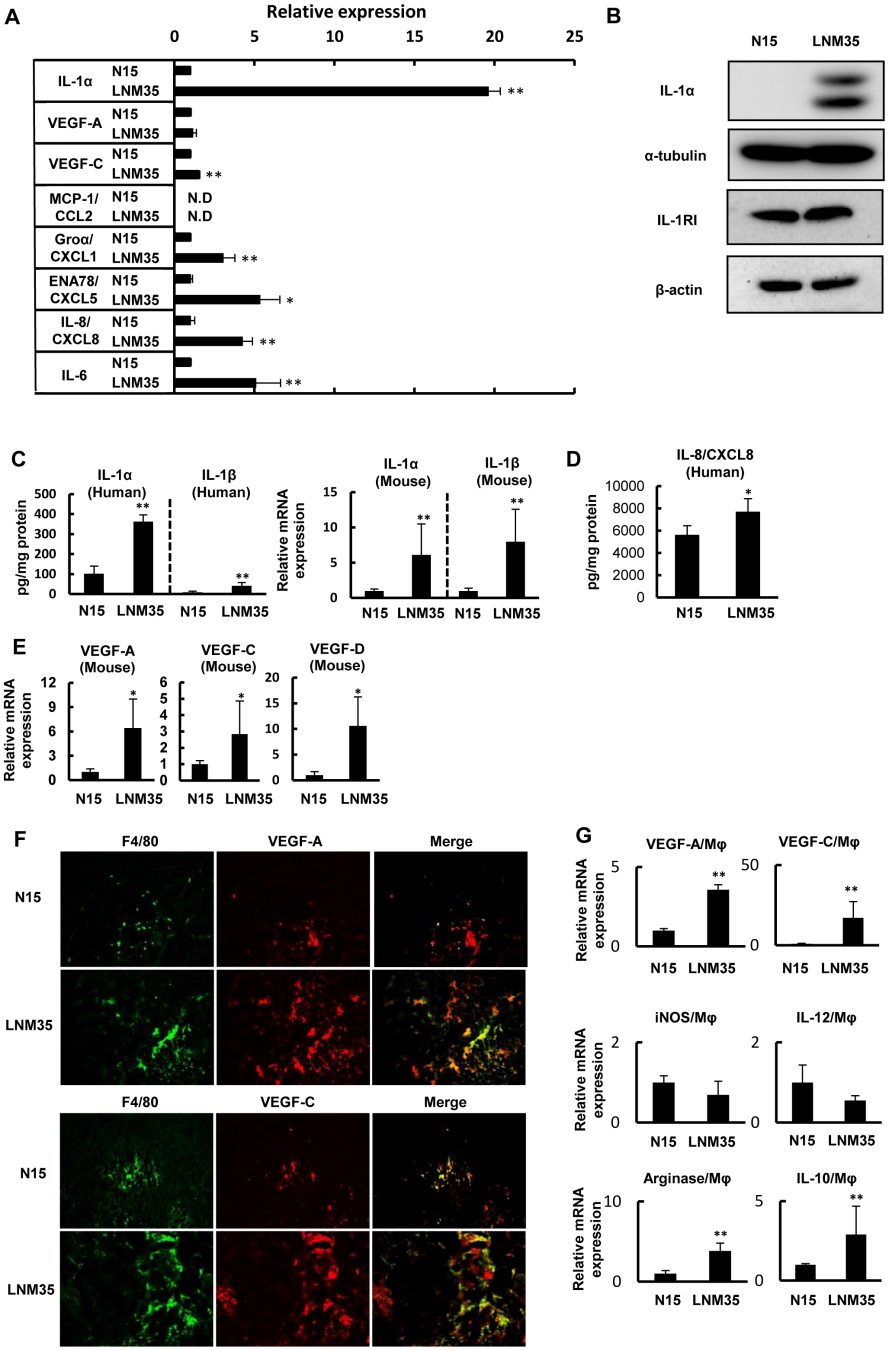
Enhanced expression of IL-1s, CXCL8/IL-8 and VEGFs in highly metastatic tumor. (A) The relative expression of IL-1α, VEGF-A, VEGF-C, MCP-1/CCL2, Groα/CXCL1, ENA-78/CXCL5, IL-8/CXCL8, and IL-6 was determined by ELISA of the culture medium of N15 and LNM35 cells. *p<0.05 and **p<0.01 indicate significant differences between N15 and LNM35 cells (triplicate cultures). The data were normalized to 1.0 based on N15 expression. (B) Western blots showing the levels of IL-1α and IL-1RI expression in LNM35 and N15 cells. (C) Comparison of human and mouse IL-1α and IL-1β protein and/or mRNA expression in N15 and LNM35 tumors. Protein expression was determined by ELISA, and mRNA levels by qRT-PCR. Six tumors for each cancer cell line were analyzed on day 35; **p<0.01. (D) Human CXCL8/IL-8 protein expression in N15 and LNM35 tumors was compared by ELISA in six tumors each on day 35; *p<0.05. (E) Mouse VEGF-A, VEGF-C, and VEGF-D mRNA expression in N15 and LNM35 tumors was compared by qRT-PCR in six tumors each on day 35; *p<0.05. (F) IHC analysis using the macrophage-specific antibody (F4/80) and human and mouse VEGF-A- or VEGF-C-specific antibodies. F4/80+ macrophages (green) colocalized with VEGF-A (red) (upper panel) and VEGF-C (red) (lower panel), as shown in yellow (merged). (G) Determination of M1-type and M2-type macrophages in tumors. VEGF-A, VEGF-C, IL-12, iNOS, IL-10, and arginase expression was determined by qRT-PCR of purified TAMs derived from both tumors. ** *p*<0.01.

In the next set of experiments, we examined the mechanisms underlying the high metastatic growth potential of LNM35 cells. Consistent with previous microarray analysis data [20], neither tumor necrosis factor-α nor macrophage-colony-stimulating factor expression was detected in our microarray analyses of LNM35 and N15 cells. By contrast, the mRNA levels of the inflammatory cytokines IL-1α and IL-1β were much higher in LNM35 cells (data not shown). The *in vitro* expression of human IL-1α, measured by ELISA, was also about 20-fold higher in the highly metastatic cells (109.13±1.9 pg/mg protein v.s. 2139.84±81.81 pg/mg protein) (Figure 2A). IL-1β protein expression was not detectable by either ELISA or western blotting (data not shown), while expression of the chemokines CXCL1/Groα (622.46±21.45 pg/ml/105 cells v.s. 1890.82±462.85 pg/ml/105 cells), CXCL5/ENA-78 (199.7±25.7 pg/ml/105 cells v.s. 1064.54±251.29 pg/ml/105 cells), CXCL8/IL-8 (195.48±51.13 pg/ml/105 cells v.s. 828.95±122.92 pg/ml/105 cells), and IL-6 (104.6±5.1 pg/ml/105 cells v.s. 531.4±161.9 pg/ml/105 cells) was three- to five-fold higher and that of VEGF-C was 1.4-fold higher in LNM35 cells (35.6±1.32 pg/ml/105 cells) than in N15 cells (23.15±0.92 pg/ml/105 cells) (Figure 2A). By contrast, there was no difference in the cellular VEGF-A levels between the two cell lines (297.05±14.66 pg/ml/105 cells v.s. 338.0±70.35 pg/ml/105 cells), and CCL2/MCP-1, a representative CC chemokine, was not detectable in either cell line by ELISA (Figure 2A). Western blot analysis showed markedly higher IL-1α expression by the highly metastatic cancer cells, while IL-1RI expression in the two cell lines was similar (Figure 2B).

### Expression of IL-1, CXCL8/IL-8, and VEGF family proteins is enhanced in highly metastatic cancer cells and tumor macrophages in vivo

We then measured the expression of IL-1 proteins (IL-1α and IL-1β), CXCL8/IL-8, and VEGF family proteins (VEGF-A, -C, and -D) in cancer cells and/or tumor stromal macrophages in lower and highly metastatic tumors. As shown in Figure 2C, LNM35 tumors expressed approximately four-fold higher levels of human IL-1α and IL-1β proteins and ten-fold higher levels of mouse IL-1α and IL-1β mRNAs than N15 tumors. LNM35 tumors also expressed about 1.4-fold higher levels of human CXCL8/IL-8 proteins than N15 tumors (Figure 2D). The expression of mouse VEGF-A, VEGF-C, and VEGF-D mRNAs was also higher in LNM35 tumors (Figure 2E), suggesting that tumor stromal cells are responsible for the increased expression of both angiogenic and lymphangiogenic factors.

Enhanced macrophage (F4/80) infiltration in LNM35 tumors was determined by IHC analysis. The majority of F4/80+ macrophages were co-immunostained with antibodies against VEGF-A and VEGF-C (Figure 2F). Consistent with these findings, the mRNAs for VEGF-A and VEGF-C were four- to six-fold higher in macrophages purified from LNM35 tumors than from N15 tumors (Figure 2G).

Activated macrophages can be classified into inflammation-stimulated M1 and M2 types [8], [18]. Therefore, we compared LNM35 and N15 TAMs using specific markers for the M1 (IL-12 and iNOS) and M2 (IL-10 and arginase) types. Compared with macrophages from lower metastatic tumors, those from highly metastatic tumors were strongly positive for IL-10 and arginase, and showed weaker expression of IL-12 and iNOS (Figure 2G). Taken together, these findings suggest that LNM35 tumor macrophages are mainly of the M2 type, and express high levels of both VEGF-A and VEGF-C.

### A macrophage-targeting drug suppresses tumor growth, angiogenesis, lymphangiogenesis, and lymph node metastasis in vivo

**Figure 3:**
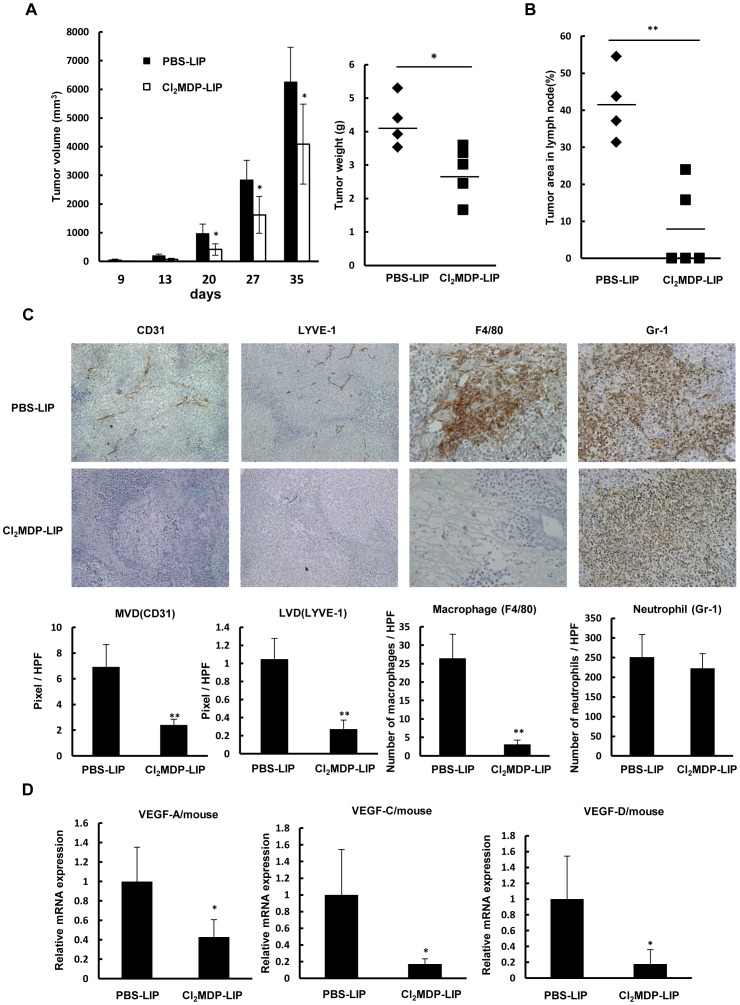
Effect of macrophage depletion on tumor growth, lymph node metastasis, and VEGF-A and VEGF-C expression. (A) Anti-tumor effect of Cl_2_MDP-LIP on tumor growth by LNM35 xenografts. Mice were subcutaneously inoculated with LNM35 cells at day 0, and tumor growth was followed until day 35 in animals intravenously injected twice weekly with PBS-LIP or Cl_2_MDP-LIP. *p<0.05 between PBS-LIP and Cl_2_MDP-LIP-treated groups (n = 5 mice per group). (B) Inhibitory effect of Cl_2_MDP-LIP on lymph node metastasis. The area occupied by cancer cells in the lymph node was determined by H&E staining. Relative tumor area is expressed as the percent of the total lymph node area (n = 5 mice per group); **p<0.01 compared with the PBS-LIP-treated group. (C) Effect of Cl_2_MDP-LIP on angiogenesis, lymphangiogenesis, and macrophage and neutrophil infiltration by LNM35 tumors. IHC analysis was performed on day 35 using specific antibodies for vascular endothelium (CD31), lymphatic vessels (LYVE-1), infiltrated macrophages (F4/80), and infiltrated neutrophils (Gr-1). Five areas of each tumor section from five tumor samples were quantitatively analyzed; **p<0.01. (D) Effect of Cl_2_MDP-LIP on the expression of mouse VEGF-A, VEGF-C, and VEGF-D mRNA in LNM35 tumors, determined by qRT-PCR analysis of five tumors on day 35; *p<0.05 compared with the PBS-LIP-treated group.

The production of angiogenic and lymphangiogenic factors by activated macrophages in highly metastatic tumors suggested that their depletion would reduce tumor angiogenesis and lymphangiogenesis. We previously demonstrated the anti-tumor and anti-angiogenic effects of the liposome-encapsulated macrophage-targeting agent bisphosphonate (Cl_2_MDP-LIP) in a xenograft model [12]–[14], [26], [27]. That study provided evidence for a pivotal role of macrophages in tumor growth, angiogenesis, and metastasis. Here, we examined the effects of Cl_2_MDP-LIP on tumor growth, lymph node metastasis, and lymphangiogenesis in highly metastatic LNM35 cells. The results showed that Cl_2_MDP-LIP significantly inhibited tumor growth, based on reductions in tumor volume and weight (Figure 3A), and significantly suppressed lymph node metastasis (Figure 3B). In IHC analyses of tumor sections, the infiltration of macrophages, but not neutrophils, and the development of lymphatic and angiogenic vessels were suppressed in the drug-treated tumors. Quantitative analyses of the Cl_2_MDP-LIP-treated tumors showed significant suppression of macrophage infiltration, angiogenesis, and lymphangiogenesis (Figure 3C), while the mouse VEGF-A, VEGF-C, and VEGF-D mRNA levels were significantly lower (*p<0.05; Figure 3D), indicating that Cl_2_MDP-LIP suppressed VEGF-A, VEGF-C, and VEGF-D expression by TAMs.

### Macrophages recruited and activated by cancer cells in vivo acquire M2-type characteristics and express VEGF-A and VEGF-C through IL-1/IL-1R signaling

**Figure 4:**
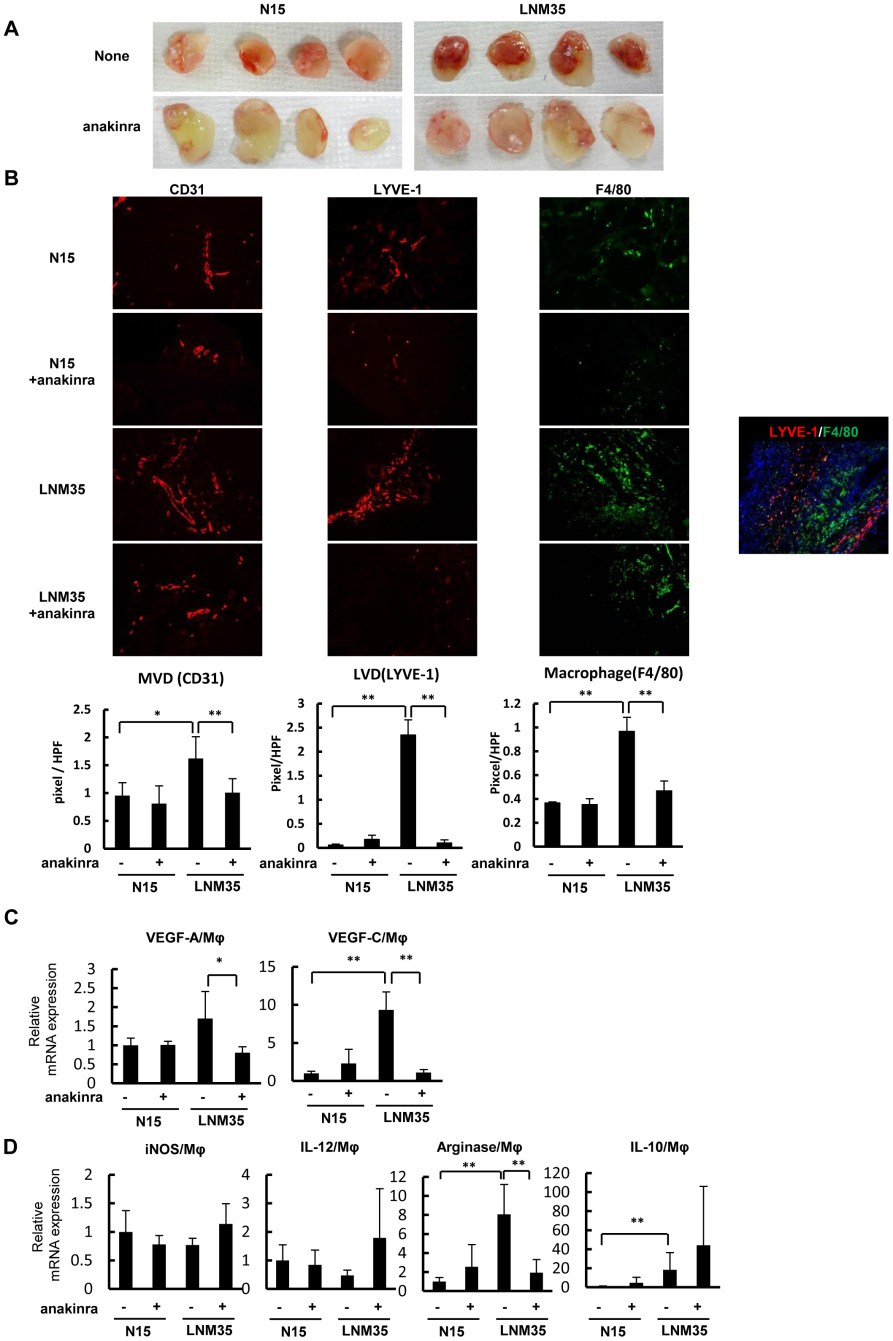
Effect of IL-1Ra on lymphangiogenesis and the accumulation of M2-type macrophages by Matrigel plug assay. (A) Matrigel plugs containing N15 and LNM35 with or without anakinra (n = 4 each). (B) Tumor angiogenesis, lymphangiogenesis, and infiltrated macrophages in Matrigel plugs (n = 6 per group) were determined immunohistochemically in frozen sections using CD31 (red), LYVE-1 (red), and F4/80 (green) as specific markers for microvessels, lymphatic vessels, and infiltrated macrophages, respectively. (C) VEGF-A and VEGF-C expression in macrophages purified from Matrigel plugs was determined by qRT-PCR. *p<0.05 and ** *p*<0.01. (D) Expression of M1- (iNOS and IL-12) and M2-type (arginase and IL-10) specific biomarkers in macrophages purified from Matrigel plugs was determined by qRT-PCR. ** *p*<0.01.

Because IL-1α expression was augmented in the highly metastatic cancer cells and tumors (Figure 2A–C), we examined whether IL-1/IL-1R signaling was involved in the high angiogenic and lymphangiogenic potential of LNM35 tumors. Matrigel plugs containing human lung cancer cells with or without the IL-1R antagonist anakinra were subcutaneously implanted in mice. The deeper red color of the untreated LNM35 plugs compared with the anakinra-treated LNM35 plugs and all of the N15 plugs suggested more intense angiogenesis through IL-1/IL-1R signaling promotion (Figure 4A). Higher microvascular (CD31+) and lymphatic vessel (LYVE-1+) densities as well as larger numbers of infiltrating macrophages (F4/80+) were found in LNM35 plugs compared with N15 plugs with or without anakinra treatment (Figure 4B). Consistent with these observations, treatment with the IL-1R antagonist significantly suppressed lymphangiogenesis, angiogenesis, and macrophage infiltration in the LNM35 plugs (Figure 4B). Co-immunostaining of these plugs for the macrophage marker F4/80 and lymphatic vessel marker LYVE-1 demonstrated that the LYVE-1+ cells were lymphatic vessels and not LYVE-1+ macrophages (Figure 4B). IHC staining for another lymphatic vessel marker, podoplanin, confirmed the presence of more intense lymphangiogenesis in the LNM35 plugs (data not shown).

To characterize the macrophages recruited and activated by cancer cells to promote angiogenesis and lymphangiogenesis, we performed experiments using Matrigel plugs containing human lung cancer cells in the presence or absence of anakinra. Macrophages that accumulated in LNM35 plugs expressed relatively higher levels of VEGF-A and VEGF-C than those that accumulated in N15 plugs with or without anakinra (Figure 4C). Anakinra markedly blocked VEGF-A and VEGF-C expression in the macrophages in the LNM35 plugs. Furthermore, the expression of arginase and IL-10 was higher and that of iNOS and IL-12 was lower in TAMs in the LNM35 plugs, but not in those in the N15 plugs (Figure 4D), suggesting that the macrophages recruited by LNM35 cells are mainly of the M2 type. Anakinra treatment of the LNM35 plugs increased the expression of iNOS and IL-12 and decreased the expression of arginase in macrophages (Figure 4D), suggesting a critical role for IL-1/IL-1R signaling in M2-type macrophage accumulation. The more abundant VEGF-A and VEGF-C expression in LNM35 TAMs indicates that these M2-type macrophages are highly angiogenic and lymphangiogenic.

To characterize the macrophages recruited and activated by highly aggressive mouse lung cancer cells, we again used a syngeneic mouse model, thus building on the results of previous studies that examined the effects of LLC/IL-1β cells and their low inflammatory counterpart LLC/neo cells expressing high and low levels of IL-1β, respectively [13], [14]. That work showed more intensive angiogenesis and tumor growth as well as enhanced macrophage accumulation in the tumor microenvironment of syngeneic mice implanted with LLC/IL-1β cells. In the present study, LLC/IL-1β Matrigel plugs contained more blood and were thus redder than LLC/neo plugs (Figure S1A). Thin sections of the plugs showed more intense angiogenesis (CD31+) and lymphangiogenesis (LYVE-1+) and an increased number of infiltrating macrophages (F4/80+) in the LLC/IL-1β plugs compared with the LLC/neo plugs (Figure S1B). Quantitative analyses of six plugs showed significant increases in the microvascular and lymphatic vessel densities and numbers of infiltrating macrophages in the LLC/IL-1β plugs (Figure S1B). VEGF-A and VEGF-C expression was also markedly higher in the macrophages in the LLC/IL-1β plugs (Figure S1C). The expression of IL-10 and arginase in the LLC/IL-1β plugs demonstrated that the macrophages were of the M2 type (Figure S1D). In addition, we found that the iNOS levels in the macrophages in the LLC/IL-1β plugs were five-fold higher than those in the LLC/neo plugs (Figure S1D), while IL-12 expression was not detected in any of the plugs (data not shown). The reasons for the up-regulation of iNOS expression in the LLC/IL-1β-associated macrophages are unclear.

### Macrophage migration and CXC chemokine expression by highly metastatic cancer cells are inhibited by the IL-1R antagonist anakinra in vitro

**Figure 5:**
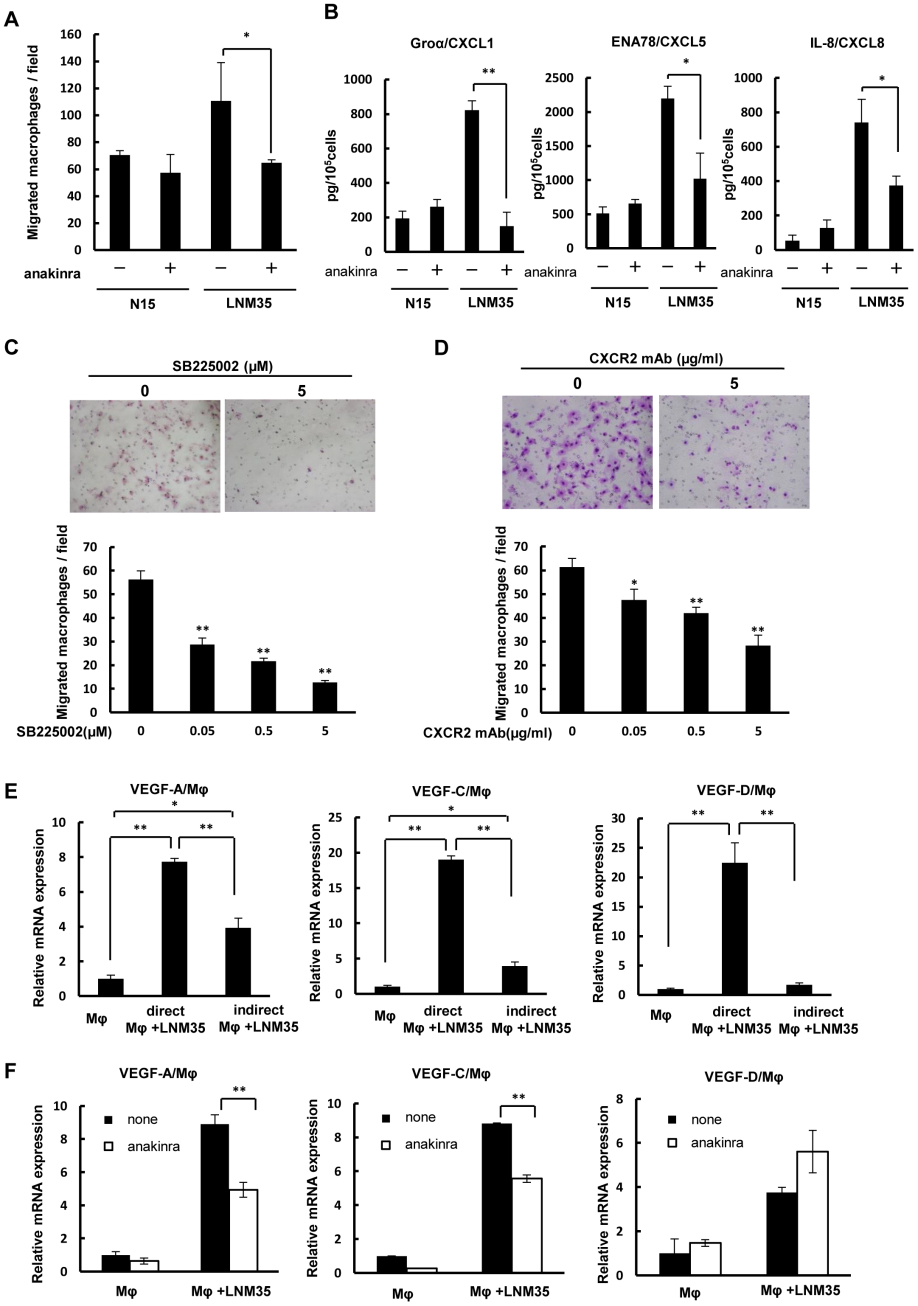
Effect of IL-1Ra on VEGFs expression by macrophages when co-cultured with cancer cells. (A) Effect of anakinra on the migration of macrophages co-cultured with N15 or LNM35 cells; *p<0.05, triplicate samples. (B) Effect of anakinra on Groα/CXCL1, ENA-78/CXCL5, and IL-8/CXCL8 expression in N15 and LNM35 cells as determined by ELISA; *p<0.05 and **p<0.01, triplicate cultures. Effect of a CXCR2 antagonist (C) and a CXCR2 neutralizing antibody (D) on macrophage migration induced by highly metastatic LNM35 cells; *p<0.05 and **p<0.01, triplicate cultures. (E) Expression of mouse VEGF-A, VEGF-C, and VEGF-D in macrophages co-cultured directly or indirectly with LNM35 cells. In indirect co-cultures, mouse macrophages were placed in the lower chamber with or without cancer cells in the upper chamber. After 4 days, mouse VEGF-A, VEGF-C, and VEGF-D mRNA expression was analyzed by qRT-PCR; *p<0.05 and **p<0.01, triplicate cultures. (F) Effect of anakinra on mouse VEGF-A, VEGF-C, and VEGF-D expression in macrophages directly co-cultured with LNM35 cells. Mouse macrophages were co-cultured with or without cancer cells for 4 days in the presence or absence of 50 ng anakinra/mL, followed by the analysis of mouse VEGF-A, VEGF-C, and VEGF-D mRNA expression by qRT-PCR; **p<0.01, triplicate cultures.

The higher number of macrophages and augmented expression of IL-1α and CXC chemokines in highly metastatic tumors versus lower metastatic tumors led us to examine whether IL-1/IL-1R and/or CXC chemokines/CXCR2 were involved in the ability of LNM35 cells to recruit macrophages. Indeed, the macrophage migration induced by these cells was two-fold higher than that by N15 cells and was largely blocked by anakinra, as determined by Boyden-chamber-based cell migration assays (Figure 5A). The increased expression of the CXC chemokines Groα/CXCL1, ENA-78/CXCL5, and IL-8/CXCL8 in the highly metastatic cells was reduced to less than half of that in the presence of anakinra (Figure 5B). Similarly, in macrophages treated with SB225002, a receptor antagonist of CXCR2, macrophage migration induced by LNM35 cells was markedly suppressed in a dose-dependent manner (Figure 5C). In addition, incubation of macrophages with a CXCR2 neutralizing antibody prevented the tumor-cell-induced macrophage migration (Figure 5D). Thus, in highly metastatic cancer cells, the enhanced expression of CXC chemokines in response to endogenous IL-1α seems to stimulate macrophage migration.

### Enhanced VEGF-A and VEGF-C expression by direct co-culture of macrophages with cancer cells is blocked by anakinra

In the xenograft model, the expression of mouse VEGF-A, VEGF-C, and VEGF-D was up-regulated in highly metastatic tumors (Figure 2E–G). Therefore, we further examined whether these effects were mediated by IL-1. In a preliminary experiment, we determined that direct co-culture of macrophages with cancer cells induced up-regulation of VEGF-A, VEGF-C, and VEGF-D in macrophages (Figure 5E) and that the up-regulation of VEGF-A and VEGF-C, but not VEGF-D, under these conditions was suppressed by anakinra (Figure 5F).

### Effects of anakinra on tumor growth and development of lymphatic and angiogenic vessels in vivo

**Figure 6:**
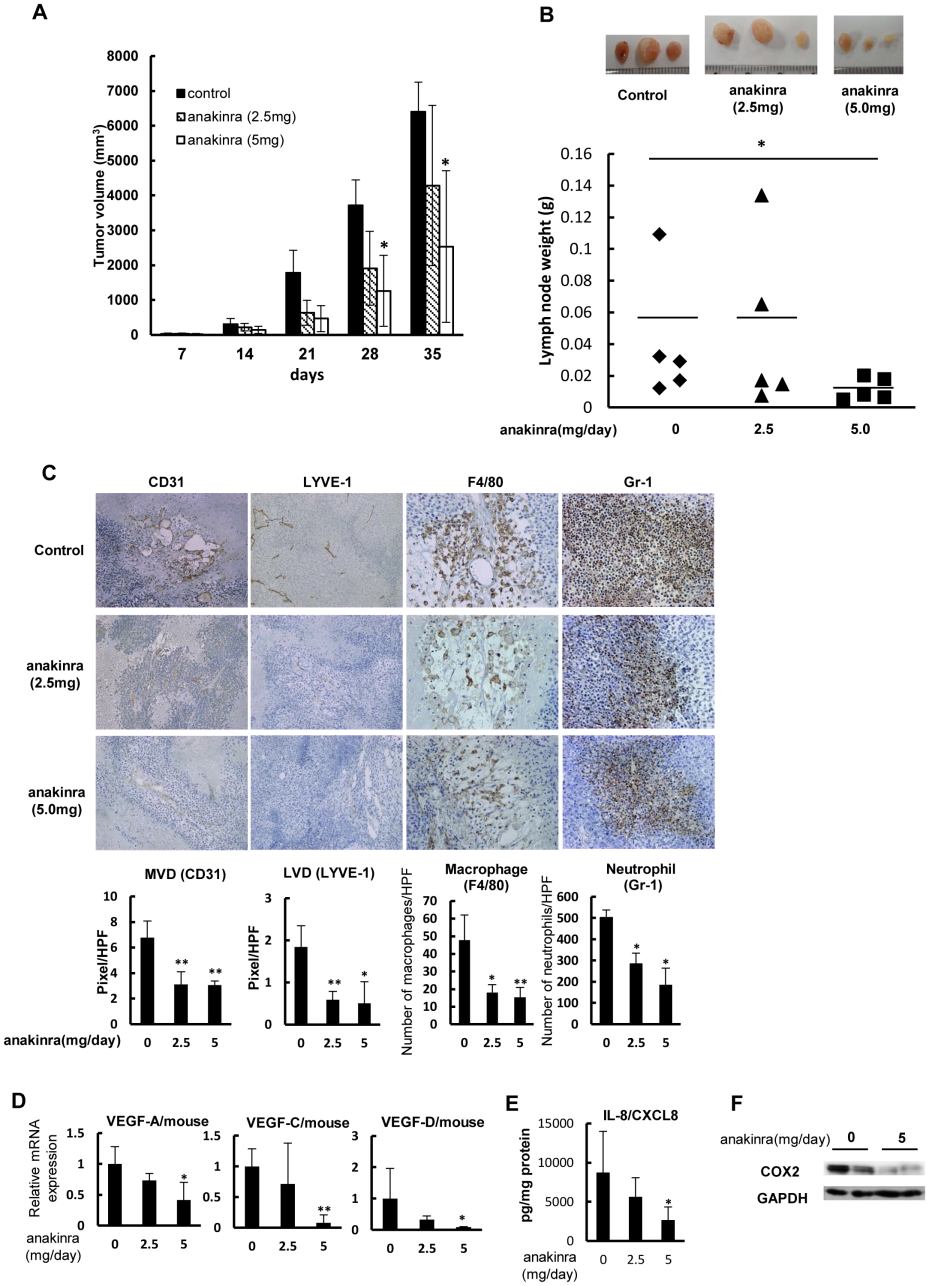
Effect of IL-1Ra on tumor growth and lymph node metastasis by highly metastatic cancer cells. (A) Anti-tumor effect of anakinra on the growth of LNM35 xenografts. LNM35 cells were subcutaneously inoculated at day 0, after which tumor growth with or without a daily subcutaneous injection of anakinra was followed until day 35; *p<0.05 between non-treated and treated groups (n = 5 mice per group). (B) Inhibition of lymph node metastasis by anakinra. Three metastatic lymph nodes representative of each group at day 35 are shown. (C) Effects of anakinra on angiogenesis, lymphangiogenesis, and macrophage and neutrophil infiltration by LNM35 tumors, analyzed on day 35 by IHC staining using specific antibodies for vascular endothelium (CD31), lymphatic vessels (LYVE-1), infiltrated macrophages (F4/80), and infiltrated neutrophils (Gr-1). Five areas of each tumor section from five tumor samples were quantitatively analyzed; *p<0.05 and **p<0.01. (D) Effect of anakinra on mouse VEGF-A, VEGF-C, and VEGF-D mRNA levels in LNM35 tumors, determined by qRT-PCR analysis of five tumors at day 35; *p<0.05 and **p<0.01. (E) Effect of anakinra on human CXCL8/IL-8 protein levels in LNM35 tumors, determined by ELISA analysis of five tumors at day 35; *p<0.05. (F) Inhibitory effect of human COX2 expression in anakinra-treated tumors. COX2 expression in two representative tumors from mice treated or not with anakinra (5 mg) was analyzed by western blotting.

Next, we examined whether the IL-1R antagonist anakinra exhibited therapeutic effects on lymph node metastasis, angiogenesis, lymphangiogenesis, and macrophage infiltration *in vivo*. In LNM35 xenograft mice, subcutaneous administration of anakinra suppressed tumor growth, although significant reductions in tumor volume were only obtained for a dose of 5 mg/day, and not 2.5 mg/day (*p<0.05; Figure 6A). Treatment with the higher dose also resulted in a significant decrease in lymph node metastasis (*p<0.05; Figure 6B).

IHC analyses revealed the development in untreated tumors of both a neovasculature (CD31+) and lymphatic vessels (LYVE-1+), as well as the infiltration of macrophages (F4/80+) and neutrophils (Gr-1+) (Figure 6C). By contrast, in anakinra-treated tumors, blood and lymphatic vessel development was reduced, as was macrophage and neutrophil infiltration. Quantitative analyses of CD31-positive and LYVE-1-positive vessels showed significant reductions in the tumor microvascular and lymphatic vessel densities in anakinra-treated tumors versus untreated tumors (*p<0.05, **p<0.01; Figure 6C). Significantly reduced levels of VEGF-A, VEGF-C, and VEGF-D mRNAs were observed in tumors treated with anakinra at 5 mg/day (*p<0.05, **p<0.01; Figure 6D). This dose also effectively blocked CXCL8/IL-8 expression in the cancer cells (Figure 6E), and COX2 expression in the tumors, as determined by western blot analyses (Figure 6F).

## Discussion

The highly metastatic human lung cancer cell line LNM35 was established more than a decade ago by selection of cells with high metastatic potential in mice. LNM35 cells express high levels of COX-2 and exhibit both activated lymphangiogenic VEGFR-3 signaling and a high rate of lymph node metastasis [20], [21]. The findings obtained in the present study shed light on the mechanisms by which LNM35 cells induce lymphangiogenesis, angiogenesis, and lymph node metastasis because all of these processes were mediated by potent, IL-1-induced, inflammatory stimuli in the tumor microenvironment. In contrast to their lower metastatic counterparts, the highly metastatic cancer cells are characterized by several properties related to malignant progression. These include: (1) augmented expression of IL-1α *in vitro* and *in vivo*; (2) higher expression levels of CXC chemokines, such as CXCL1/Groα, CXCL5/ENA-78, and CXCL8/IL-8 *in vitro* and *in vivo*; (3) CXCR2-mediated increase in macrophage migration; (4) *in vivo* recruitment of M2-type macrophages expressing VEGF-A and VEGF-C to the tumor stroma and, in Matrigel plug assays, recruitment and activation of these cells by either syngeneic mouse cancer cells expressing IL-1β or heterogeneic cancer cells expressing IL-1α; and (5) significant suppression by the IL-1R antagonist anakinra of macrophage infiltration, lymphangiogenesis, and angiogenesis in tumor stroma, and lymph node metastasis. Taken together, our results provide strong evidence for the pivotal role of IL-1/IL-1R signaling in tumor lymphangiogenesis, tumor angiogenesis, and lymph node metastasis.

**Figure 7:**
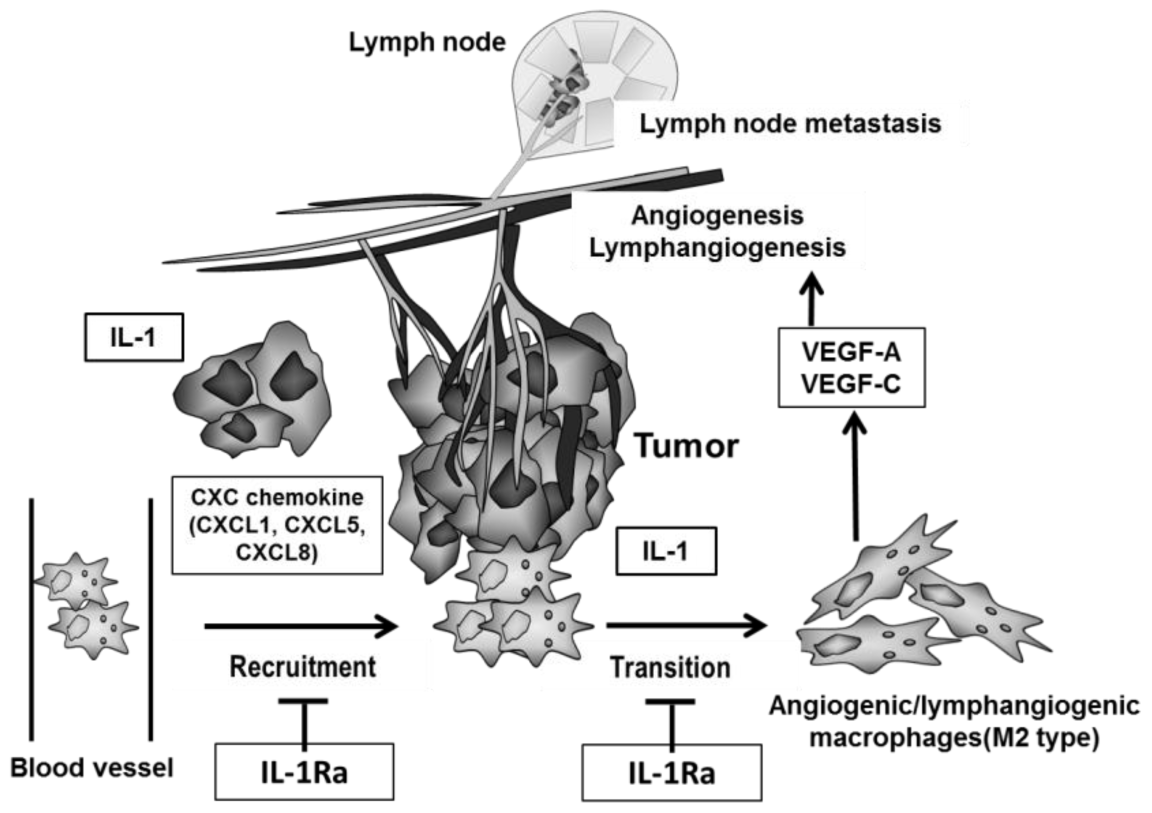
Our hypothetical model how macrophages and cancer cells mutually promote lymph node metastasis through IL-1/IL-1R. Lung cancer cells form lymph node metastases, promote the development of lymphatic vessels, exhibit invasive tumor growth, and constitutively express IL-1 and CXC chemokines. Highly metastatic cancer cells intrinsically produce larger amounts of IL-1 and CXC chemokines. IL-1α and, to a lesser extent, IL-1β induce the up-regulation of CXC chemokines, which in turn promotes the recruitment and activation of macrophage in the tumor microenvironment. Direct interaction of these cancer cells with these macrophages markedly enhances the production of potent angiogenic and lymphangiogenic factors, leading to further malignant progression including lymph node metastasis and lymphangiogenesis. This sequence of events is suppressed by the IL-1R antagonist anakinra.

Previous studies have shown that infiltration of macrophages in the tumor microenvironment supports tumor growth, angiogenesis, inflammation, metastasis/invasion, and immunosuppression through the production of factors promoting tumor progression [6], [8]–[11], [18], [28]. The presence and activities of these factors reflect the mutual interactions of cancer cells and monocytes/macrophages under inflammatory stimuli *in vitro* and *in vivo* (Figure 7). Macrophages that support aggressively malignant tumors are mainly of the M2 type. The importance of specialized cell subpopulations, such as metastasis-associated, angiogenic, and invasive macrophages, in cancer progression suggests their appropriateness as therapeutic targets [11]. Support for this approach is provided by our results with anakinra, which suppressed the macrophage-supported tumor growth, angiogenesis, lymphangiogenesis, and lymph node metastasis induced by highly metastatic cancer cells (Figures 4 and 6).

Moreover, the increased expression of IL-1α, IL-1β, VEGF-A, VEGF-C, and VEGF-D in tumor stromal cells, including macrophages (Figure 2C and E–G), suggests a mechanism by which tumor growth, angiogenesis, lymphangiogenesis, and lymph node metastasis are enhanced in highly metastatic cancer cells. Macrophages that accumulated in the tumor stroma were mainly of the M2 type. These cells expressed both angiogenic and lymphangiogenic factors and accordingly promoted the respective processes in Matrigel plugs containing highly aggressive and inflammatory cancer cells (Figure 4). Similar results for lymphangiogenesis, angiogenesis, and infiltration of M2-type macrophages expressing VEGF-A and VEGF-C were obtained in the syngeneic mouse model (Figure S1). These findings suggest a sequence of events in which macrophages are activated by the inflammatory stimuli of malignant cancer cells and become able to induce angiogenesis, lymphangiogenesis, and lymph node metastasis.

The inflammatory cytokine IL-1α is mainly localized intracellularly and on the cell membrane, while IL-1β is secreted [29], [30]. Knockdown of IL-1 and/or its receptors (IL-1RI/II) was previously shown to markedly suppress tumor growth and angiogenesis [30]. Our own studies demonstrated the essential roles of IL-1α/β in both of these processes as well as in lymphangiogenesis [12]–[15], [25], [31]. Here, we have shown the greatly enhanced expression of IL-1α in highly metastatic tumors, whereas IL-1β expression was only one-tenth that of IL-1α (Figure 2C). In the stroma of the tumors, high levels of VEGF-A, VEGF-C, and VEGF-D, mainly from macrophages, were detected.

Our study points to a link between IL-1-induced inflammatory stimuli and macrophage activation for lymphangiogenesis and lymph node metastasis, in which IL-1/IL-1R provides a microenvironment favorable for malignant tumor progression by highly metastatic cancer cells *in vivo* by inducing the transition of macrophages to the M2 type. In turn, these cells promote angiogenesis and lymphangiogenesis (Figure 7). Evidence for this sequence of events is as follows. First, targeting of IL-1R with anakinra inhibited both the migration of macrophages co-cultured with highly metastatic cancer cells and the expression of VEGF-A and VEGF-C, but not that of VEGF-D (Figure 5A, F). Second, the high-level *in vitro* expression of Groα/CXCL1, ENA-78/CXCL5, and IL-8/CXCL8 in LNM35 cells was blocked in the presence of anakinra (Figure 5B). Third, inhibition of signaling by CXCR2, the cognate receptor of these cytokines, suppressed the migration of macrophages co-cultured with highly metastatic cancer cells (Figure 5C, D). Finally, *in vivo* experimental therapeutic models showed that anakinra suppressed tumor growth, lymph node metastasis, angiogenesis, lymphangiogenesis, and M2-type macrophage infiltration, accompanied by significantly reduced expression of VEGF-A, VEGF-C, and VEGF-D (Figures 4 and 6).

A number of previous studies have reported that IL-1 is involved in tumor growth, angiogenesis and metastasis. Voronov and colleagues have reported IL-1α and IL-1β have distinct effects at tumor sites, using genetically engineered cells or mice with distinct patterns of IL-1 expression [32]. They showed that tumor cell- or host-derived IL-1β promoted tumor initiation, invasiveness, immunosuppression and angiogenesis while IL-1α preferentially activated anti-tumor cell immunity [32]. On the other hand, some reports have demonstrated that IL-1α is served as a tumor promoter and poor prognosis factor in various tumors. Ling et al. (2012) have shown a key role of IL-1α in progression of Kras^G12D^ mutation-induced pancreatic ductal adenocarcinoma in mice. This study shows that IL-1α expression is induced by AP-1-activation by Kras^G12D^ mutation, and IL-1α and p62 positive feedback loops activates IKKβ/NF-κB signaling which activates inflammatory and proliferative responses [33]. In addition, IL-1α overexpression is correlated with Kras mutation and NF-κB activation in human pancreatic ductal adenocarcinoma specimens and poor survival in pancreatic ductal adenocarcinoma patients [33], suggesting that NF-κB activation by IL-1α/IL-1R signal is important to tumor progression. Consistent with this study, our present study demonstrated that treatment with SN-50, inhibitor of NF-κB nuclear translocation, blocked expression of Groα/CXCL1, ENA78/CXCL5 and IL-8/CXCL8 in LNM35 cells in culture (data not shown). Moreover, SN-50 significantly blocked expression of mouse VEGF-A and VEGF-C from macrophages, when co-cultured with LNM35 cells (data not shown). Our recent study has also reported that human gastric cancer cells overexpressing NDRG1 induces enhanced expression of IL-1α through activation of JNK and AP-1 (Jun/Fos), resulted in promoted expression of angiogenesis-related factors and tumor angiogenesis accompanied by macrophage infiltration [25]. In light of these studies, IL-1α may support tumor progression depending on tumor types and their malignant properties.

Furthermore, NF-κB activation may play an important role in the IL-1-induced M2-type macrophage polarization. Hagemann and colleagues (2008) have reported that IL-1R/MyD88/IKKβ signal induced M2-type polarization of bone marrow derived macrophages (BMDMs), and also that transferred BMDMs from MyD88 or IL-1R deficient mice into tumor-bearing mice significantly suppressed tumor growth than those from wild type mouse. Moreover, the resident ascitic CD11b+ cells expressed higher levels of IL-12p40 (M1-type marker) and lower levels of arginase-1 (M2-type marker) in mice treated with MyD88 or IL-1R deficient macrophages than control groups [34]. Together, we conclude that IL-1/IL-1R signal in macrophages induces, if not all, M2-type polarization through activation of IKKβ/NF-κB pathway.

Angiogenic VEGF-A and lymphangiogenic VEGF-C and VEGF-D were up-regulated in the tumor stromal macrophages of highly metastatic tumors expressing IL-1α and/or IL-1β. VEGF-C promotes lymphangiogenesis and metastasis, including that to lymph nodes [35], [36], and its expression together with that of its receptor is closely associated with lymph node metastasis and tumor progression in lung cancer cells [37]. The expression of VEGF-C is highly sensitive to IL-1 and other inflammatory stimuli, while VEGF-D expression is stimulated by cell-cell contacts through a mechanism involving cadherin family proteins [38]–[41]. Consistent with these studies, VEGF-D expression was similarly augmented in macrophages co-cultured with lower and highly metastatic cancer cells (unpublished data), could not be induced by indirect co-culture of macrophages and cancer cells (Figure 5E), and was not inhibited by the IL-1R antagonist anakinra. As indicated earlier, IL-1α is mainly non-secretory, while IL-1β is secretory. The importance of non-secreted IL-1α, and thus of cell-cell contacts, for VEGF-A and VEGF-C expression was also demonstrated, because the levels of these factors were much higher in direct, rather than indirect, co-cultures of macrophages and tumor cells (Figure 5E).

In conclusion, our study suggests that inflammatory stimuli contribute to the establishment of a tumor microenvironment that, through macrophage activation, enables the growth, invasion, and metastasis of cancer cells. Specifically, IL-1-driven inflammatory stimuli in cancer cells enhance lymphangiogenesis and angiogenesis through up-regulation of CXC chemokines, VEGF-A, and VEGF-C, and inducing the transition of macrophages into the lymphangiogenic M2 type. Blocking of these inflammatory stimuli with the IL-1R antagonist anakinra consequently suppresses lymph node metastasis and tumor growth by highly aggressive cancer cells. In fact, clinical studies with anakinra have been now applied to treat with breast cancer, metastatic colorectal cancer, pancreatic cancer, and multiple myeloma. A number of previous studies have reported that high levels of IL-1 in lung cancer patients were correlated with poor prognosis and metastasis. However, clinical trials with anakinra in lung cancer have never been made so far. We further expect its clinical trial for patients with metastatic lung cancer.

## Supporting Information

Figure S1 **Determination of the biological and biochemical characteristics of macrophages in the tumor microenvironment of highly angiogenic mouse cancer cells expressing high levels of IL-1β.** (A) Matrigel plugs containing LLC/neo and LLC/IL-1β cells (n = 6 per group). (B) Tumor angiogenesis, lymphangiogenesis, and macrophage infiltration in each Matrigel plug were determined immunohistochemically using specific markers for microvessels (CD31), lymphatic vessels (LYVE-1), and infiltrated macrophages (F4/80). (C) VEGF-A and VEGF-C expression in macrophages purified from Matrigel plugs, determined by qRT-PCR. (D) Expression of specific biomarkers for M1- (iNOS) and M2- (IL-10 and arginase) type macrophages purified from Matrigel plugs, determined by qRT-PCR.
(TIF)
