# Supplementary figures and images for: Tumor-Derived Interleukin-1 Promotes Lymphangiogenesis and Lymph Node Metastasis through M2-Type Macrophages
Source: PLoS One. 2014 Jun 12;9(6):e99568. doi: 10.1371/journal.pone.0099568 (PMC4055709; doi:10.1371/journal.pone.0099568)

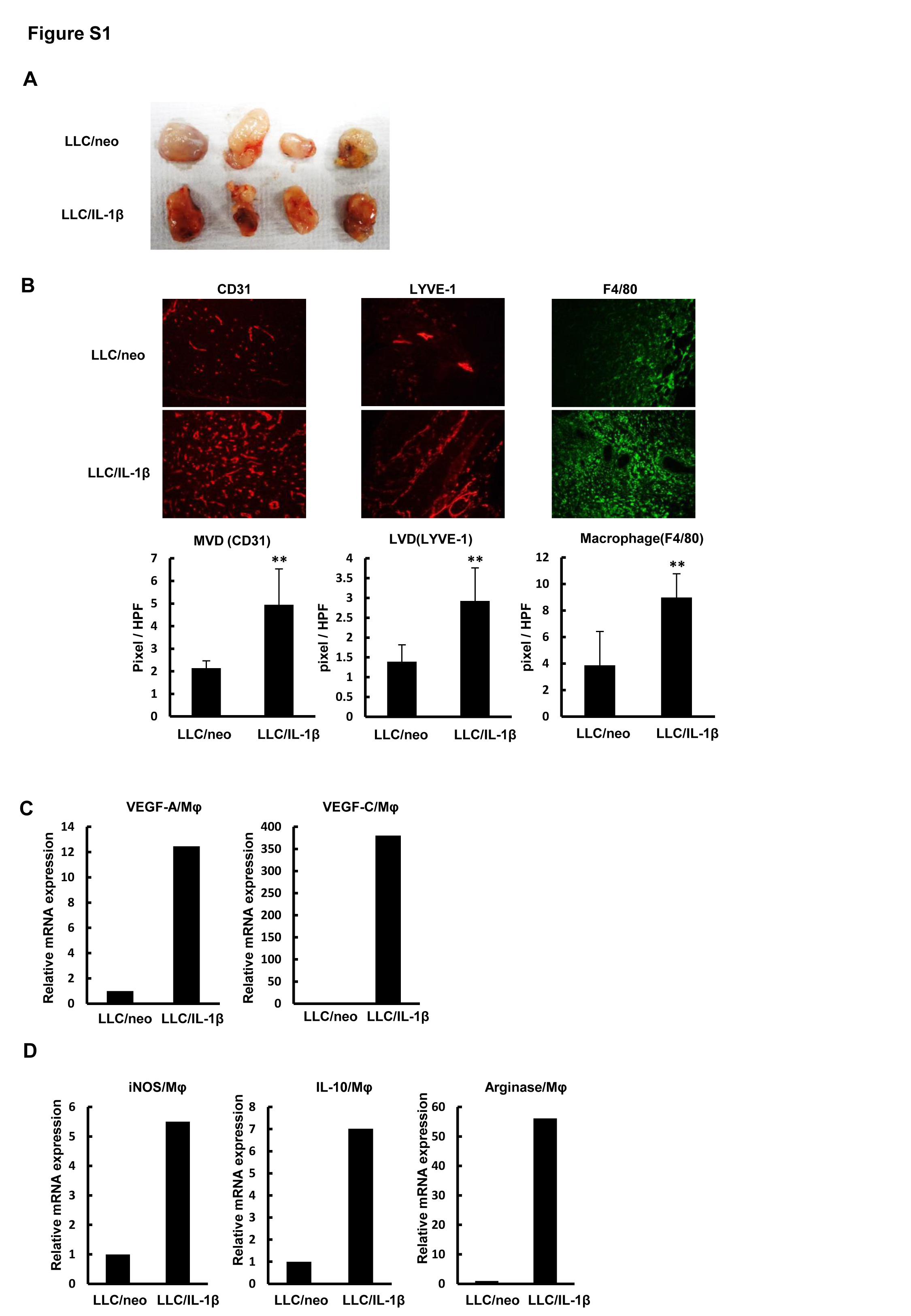

Supplement: Figure S1 — Determination of the biological and biochemical characteristics of macrophages in the tumor microenvironment of highly angiogenic mouse cancer cells expressing high levels of IL-1β. (A) Matrigel plugs containing LLC/neo and LLC/IL-1β cells (n = 6 per group). (B) Tumor angiogenesis, lymphangiogenesis, and macrophage infiltration in each Matrigel plug were determined immunohistochemically using specific markers for microvessels (CD31), lymphatic vessels (LYVE-1), and infiltrated macrophages (F4/80). (C) VEGF-A and VEGF-C expression in macrophages purified from Matrigel plugs, determined by qRT-PCR. (D) Expression of specific biomarkers for M1- (iNOS) and M2- (IL-10 and arginase) type macrophages purified from Matrigel plugs, determined by qRT-PCR. (TIF) [file pone.0099568.s001.tif]
